# Polymeric nanoparticles loaded with vincristine and carbon dots for hepatocellular carcinoma therapy and imaging

**DOI:** 10.1038/s41598-024-75332-1

**Published:** 2024-10-18

**Authors:** Walaa Fawaz, Abdulsamie Hanano, Hossam Murad, Amal Yousfan, Ibrahim Alghoraibi, Jameela Hasian

**Affiliations:** 1 Department of Pharmaceutics and Pharmaceutical Technology, Faculty of Pharmacy, Damascus University, Damascus, Syria; 2 Department of Molecular Biology and Biotechnology, Atomic Energy Commission of Syria (AECS), Damascus, Syria; 3 Department of Pharmaceutics and Pharmaceutical Technology, Faculty of Pharmacy, Al Andalus University for Medical Sciences, Tartus, Syria; 4 Department of Physics, Faculty of Science, Damascus University, Damascus, Syria; 5 Department of Pharmaceutics and Pharmaceutical Technology, Faculty of Pharmacy, Yarmouk Private University, Damascus, Syria

**Keywords:** Liver cancer, Carbon dots, Vincristine sulfate, Polycaprolactone, Nanoparticles, Bioimaging, Cancer, Materials science, Nanoscience and technology

## Abstract

Chemotherapy for hepatoblastoma is limited by organ toxicity and poor outcomes, prompting the search for new, more effective treatments with minimal side effects. Vincristine sulfate, a potent chemotherapeutic, faces challenges due to P-glycoprotein-mediated resistance and its systemic toxicity. Nanoparticles offer a promising solution by improving pharmacokinetics, targeting tumor cells, thus reducing side effects. Moreover, the use of fluorescent nanomaterials is emerging in biomedical applications such as bioimaging, detection and therapies. This study describes a promising delivery system utilizing carbon dots encapsulated with vincristine in biodegradable polycaprolactone nanoparticles via a double emulsion technique. The fine characterization of these nanoparticles showed that they are spherical, uniformly sized with around 200 nm and exhibit excellent colloidal stability. Moreover, the release profile showed prolonged release for both vincristine and carbon dots. In vitro cell viability studies revealed enhanced cancer cell inhibition for the encapsulated drug compared to the vincristine solution. The uptake study indicated clear fluorescence for carbon dots solution and vincristine and carbon dots loaded nanoparticles upon excitation. Additionally, studies on primary mouse hepatocytes demonstrated higher fluorescence intensity in treatment groups. These results suggest that vincristine and carbon dots loaded nanoparticles are effective, target-specific carriers for liver cancer treatment. Furthermore, the carbon dots were not cytotoxic, highlighting their potential in bioimaging and cancer cell studies.

**Supplementary Information:**

The online version contains supplementary material available at 10.1038/s41598-024-75332-1.

## Introduction

Hepatocellular carcinoma (HCC) is among the leading causes of cancer-related deaths worldwide^1^. It typically develops in patients with chronic hepatitis, including viral, alcoholic, and non-alcoholic hepatitis^2,3^, through a multi-step process involving complex genetic alterations and mutations, leading therefore to activate molecular signaling pathways related to cell proliferation and the avoidance of programmed cell death^4^. Although surgical resection remains the standard curative treatment for HCC, most patients are not eligible for this procedure due to advanced tumor extension at the time of diagnosis and/or insufficient liver functional reserves^5^. Furthermore, chemotherapy regimens for suitable candidates are often constrained by major organ damage, inefficient drugs, and poor prognosis. Consequently, the search for novel anticancer agents or regimens with higher efficacy and minimal side effects continues^6^. Vincristine sulfate (VRC), a highly potent chemotherapeutic agent, has been widely used in the treatment of various types of cancer^7,8^. However, its effectiveness in cancer therapy is often hindered by the overexpression of P-glycoprotein and the associated dose-limiting systemic toxicity side effects^9,10^. Nanoparticles offer numerous advantages in cancer treatment, including improved pharmacokinetics, precise targeting of tumor cells, reduced side effects, and decreased drug resistance^11^. Fluorescent nanoparticles exhibit targeted therapy capabilities and multifunctional properties^12^. One of the latest discovered fluorescent nanoparticles, carbon dots (c-dots) show great promise as bioimaging agents^13^ by overcoming the toxicity of heavy metals found in traditional semiconductor quantum dots used in the biomedical field^14^. Folic acid-derived c-dots can selectively stain cancer cells by targeting the folate receptors mainly expressed on the cell surface^15,16^. To facilitate the delivery of chemotherapy drugs, a nano-sized drug delivery system based on carbon dots was developed through surface modification or by coupling chemotherapy drugs^17^.

Several studies have utilized fluorescent carbon dots in nano drug delivery systems, demonstrating their potential for efficient emission-based delivery mechanisms^18,19^. Significant efforts have focused on developing nanocarriers that combine cell targeting with efficient in vivo imaging, controlled drug release, and detecting and isolating cancer cells^15,20,21^. Additionally, PLGA nanoparticles have been engineered to encapsulate both vincristine sulfate and verapamil, offering the potential to overcome multidrug resistance and improve therapeutic outcomes^22^. Furthermore, a targeted delivery system using doxorubicin-loaded PCL nano-capsules was developed via a double emulsion technique, enhancing the drug’s efficacy against hepatocellular carcinoma^23^.

In the current study, Vincristine sulfate nanoparticles based on carbon dots were developed using the double emulsion method, consisting of a complex system where the droplets of the dispersed phase contain intern smaller droplets and offer excellent potential for encapsulating hydrophilic substances that often exhibit low encapsulation efficiency in single emulsions due to rapid drug partitioning into the external aqueous phase^24,25^. However, the main challenge in using double emulsions lies in achieving precise control and uniform droplet size during production^26^. This necessitates the optimization of several process variables to attain a well-controlled and homogeneous droplet size distribution^27,28^. Polycaprolactone (PCL), a biodegradable polymer, was used as a nanoparticle carrier. PCL is known for its significant potential in controlled drug delivery due to its biocompatibility^29–31^. The prepared nanoparticles were evaluated for particle morphology, average dynamic size, zeta potential, encapsulation efficiency, and release profile. To explore the antitumor activity of VRC and c-dots loaded nanoparticles in cancer therapy, in vitro cytotoxicity was evaluated in HepG2 cells, and the cellular uptake was studied using inverted fluorescence microscopy. Additionally, cellular uptake was assessed in primary hepatocytes isolated from mice using fluorescence spectroscopy.

## Results and discussion

### Method of VRC and c-dots quantification

**Fig. 1:**
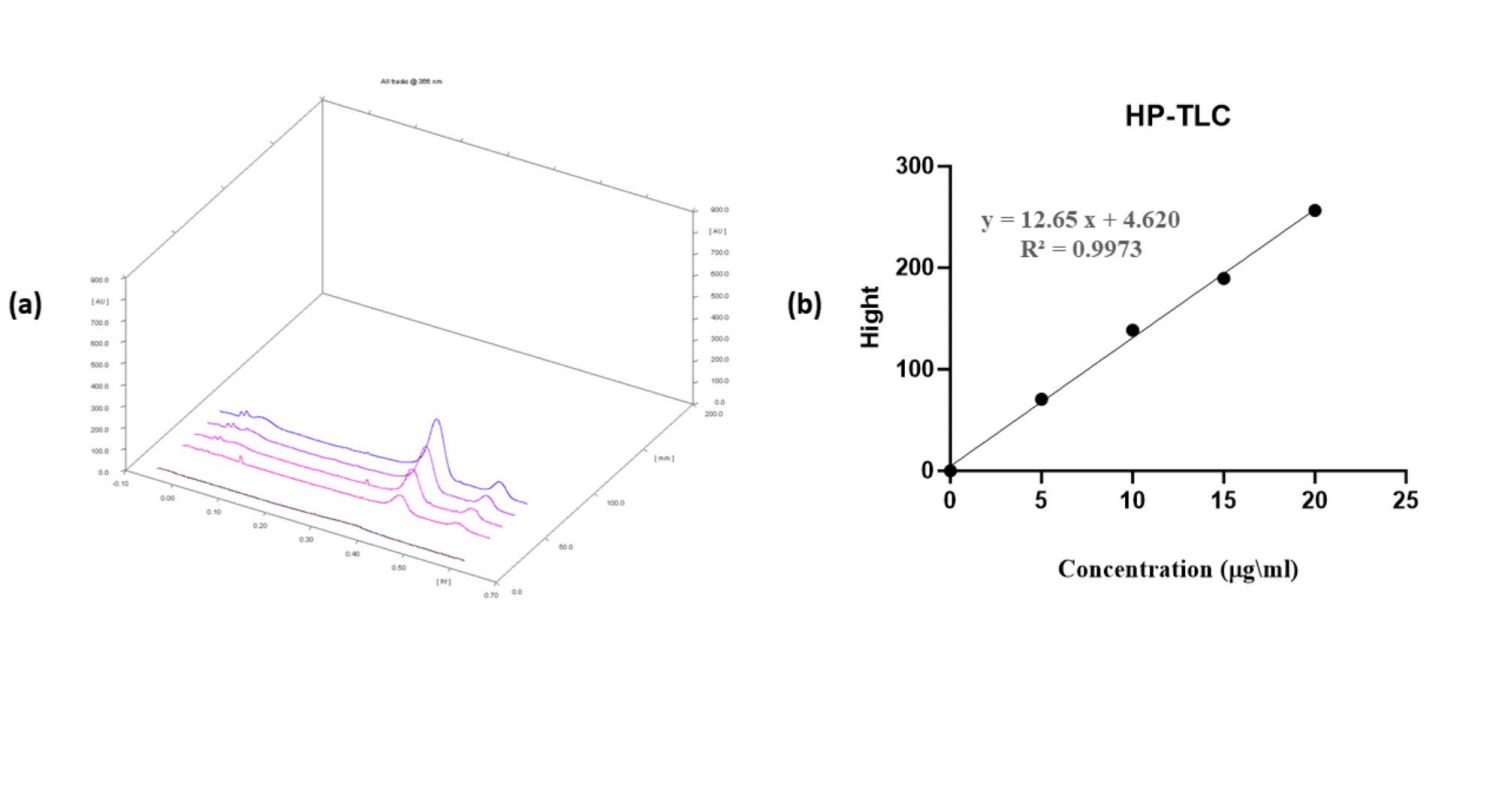
The chromatographs (**a**), and the calibration curves of carbon dots concentration (**b**) using HP-TLC.

The relationship between peak heights and carbon dots concentrations was determined using High-Performance Thin-Layer Chromatography (HP-TLC) as shown in Fig. 1. The chromatograms show the peaks corresponding to c-dots appeared symmetrical and well-separated with *Rf* value of 0.4 (Fig. 1a). A linear correlation between the peaks high and the respective concentrations, ranging from 0 to 20 µg/ml, was established (Fig. 1b).

### The preparation and physicochemical characterization of vincristine sulfate and carbon dots loaded polycaprolactone nanoparticles

VRC and c-dots loaded NPs were successfully prepared using the double emulsion technique. Carbon dots and VRC exhibit remarkable solubility in aqueous solution, making the double emulsion the most suitable method^24,32^. The nanoparticles were characterized in terms of average particle size, size distribution, surface charge, and encapsulation efficiency (Table 1). The morphological study of the prepared nanoparticles was determined using a scanning electron microscope, and the release profile of the prepared nanoparticles was examined.

**Table 1:** Size, polydispersity index (PDI), zeta potential, and encapsulation efficiency values (EE%) of the prepared VRC and c-dots loaded NPs (mean ± SD; *n* = 3).

Formulation	Zeta average (nm)	PDI	Zeta potential (mV)	EE% _VRC_	EE% _c−dots_
VRC -C- NPs 1	214.4	0.245	-23.9	36.38 ± 0.93	13.84 ± 2.44
VRC -C- NPs 2	240	0.285	-24.4	40.14 ± 1.83	25.97 ± 3.01
VRC -C- NPs 3	275	0.389	-31.9	49.87 ± 1.96	37.74 ± 2.87

**Fig. 2:**
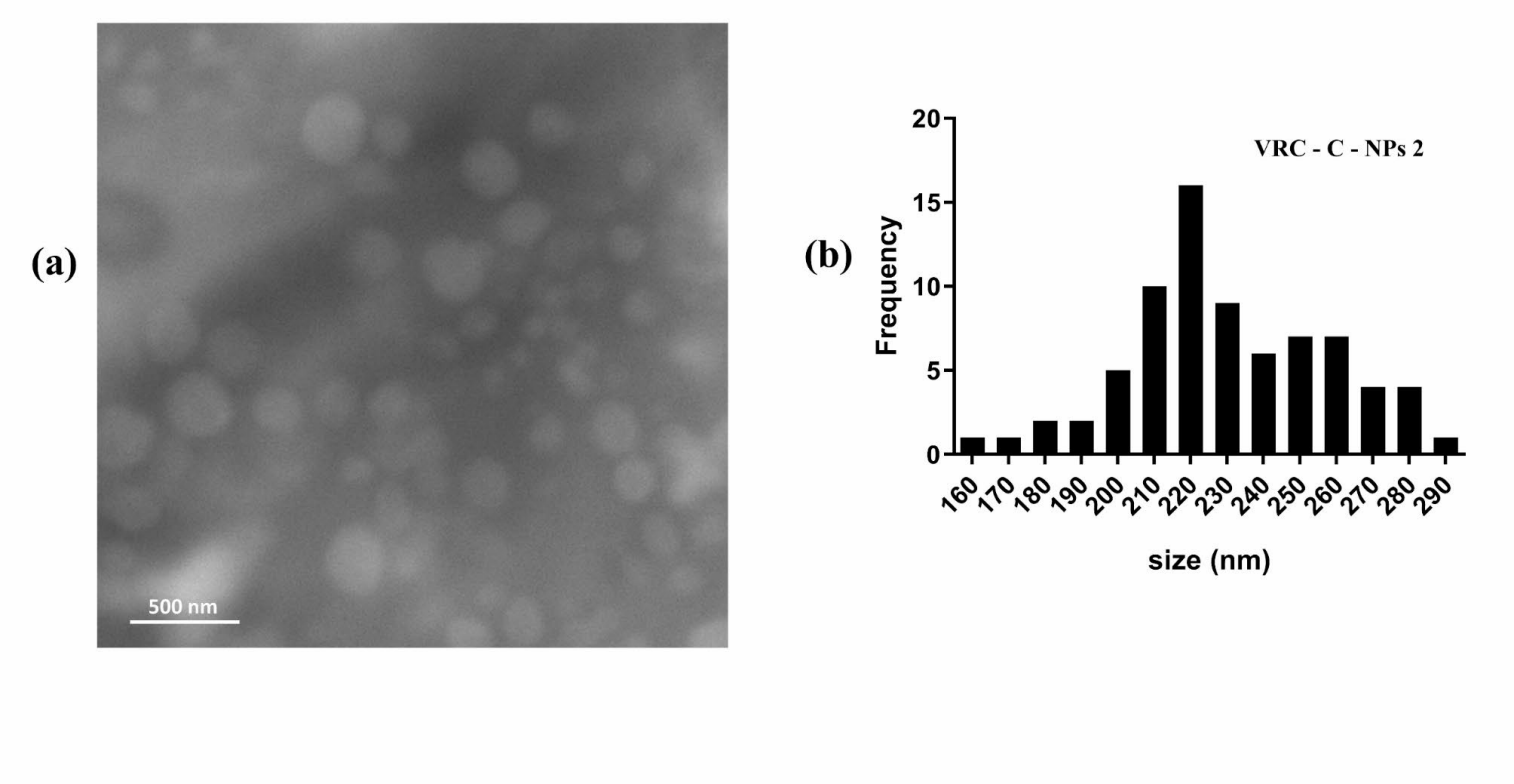
SEM micrograph of nanoparticles (**a**), and SEM size distribution histogram (**b**).

The prepared nanoparticles had an average size between 214 and 275 nm, depending on the amount of polycaprolactone (PCL) used in the preparation. This aligns with previous studies reporting that nanoparticles 200 nm in size showed extended circulation times, enhanced accumulation in tumor masses, and reduced systemic elimination^33^. The size distribution values, were less than 0.5 for all formulations, indicating acceptable homogeneity^34^. The zeta potential value (ZP) of the nanoparticles was negative, attributed to the carboxylic groups in the PCL^35,36^. It is well-recognized that ZP values can be used to evaluate the charge stability of a dispersed system. Where nanoparticles with ZP values equal to or less than − 25 mV typically exhibit a high degree of stability^37,38^. The SEM micrographs, shown in Fig. 2(a), illustrate the spherical shape of the nanoparticles, aligning with the conventional tendency that most nanocarriers are produced in spherical form for anticancer drugs. This spherical shape is crucial as it enhances the circulation time, biodistribution, cellular uptake, and targeting delivery of cancer drug^33^. Furthermore, the average particle size for formulation (VRC -C- NPs 2) was approximately 231 nm, as shown in Fig. 2(b).

**Fig. 3:**
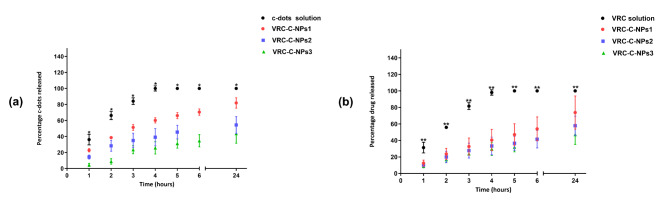
The release profile of carbon dots (**a**) and vincristine (**b**) from NPs. The results are represented as (mean% ± SD) ^*^P < 0.05, ^**^P > 0.01.

The encapsulation efficiency for c-dots ranged from 13.84 to 37.74%, increasing with the amount of polymer used in the preparation. The release of carbon dots from polycaprolactone nanoparticles was studied in comparison with a free aqueous solution of carbon dots, as shown in Fig. 3. The results were expressed by plotting the cumulative release curve over time.

The VRC-C-NPs showed a prolonged release of c-dots compared to the free solution, with around 84% of c-dots released from the aqueous solution after three hours. In contrast, 81.9%, 54.3%, and 43.5% of c-dots were released from the VRC-C-NPs 1, VRC-C-NPs 2, and VRC-C-NPs 3 formulations, respectively, after 24 h. The release of c-dots was notably higher in NPs containing the lowest amount of PCL at all measured time points. This statement confirms that the quantity of PCL used influenced the release rate from the NPs^39^. The release of c-dots from NPs exhibited an initial burst release followed by sustained release. The initial burst release was due to the rapid dissolution and release of the substance adsorbed on the nanoparticle’s surface, followed by a slow and sustained release from the core of the polymer matrix^40,41^. However, c-dots did not affect the encapsulation efficiency or the release of VRC from the nanoparticles. The encapsulation efficiency (EE%) for VRC ranged from 36.38 to 49.87%, regarding the release profile, about 98% of VRC was released from the VRC solution after 4 h, while 73%, 57%, and 47% were released from the VRC -NPs 1, VRC -NPs 2, and VRC -NPs 3 formulations, respectively, after 24 h, as noted in a previous study^42^. Subsequently, the VRC-C-NPs 1 formulation was selected for the viability and uptake assay due to its particle size of approximately 200 nm and a PDI of 0.2.

Furthermore, to determine the stability of prepared VRC and c-dots-loaded PCL nanoparticles in aqueous media containing serum proteins, Scanning Electron Microscopy was used to assess changes in particle size, size distribution, and morphology of polycaprolactone nanoparticles upon incubation with Human Serum Albumin HSA (20%) for 6 h at 37 °C. The SEM micrographs (Fig. 1S, in the supplementary information) showed no significant changes in size or morphology, with a mean nanoparticle size of 210 nm. According to the literature, the charge and hydrophobicity of nanoparticles are key factors determining the identity and quantity of biomolecules involved in forming the hard protein corona. Nanoparticles with negatively charged carboxylic acid groups (carboxyl-NPs) exhibit good stability, as no aggregation is observed due to sufficient electrostatic repulsions^43,44^.

### FTIR and UV-Vis spectroscopy analysis

**Fig. 4:**
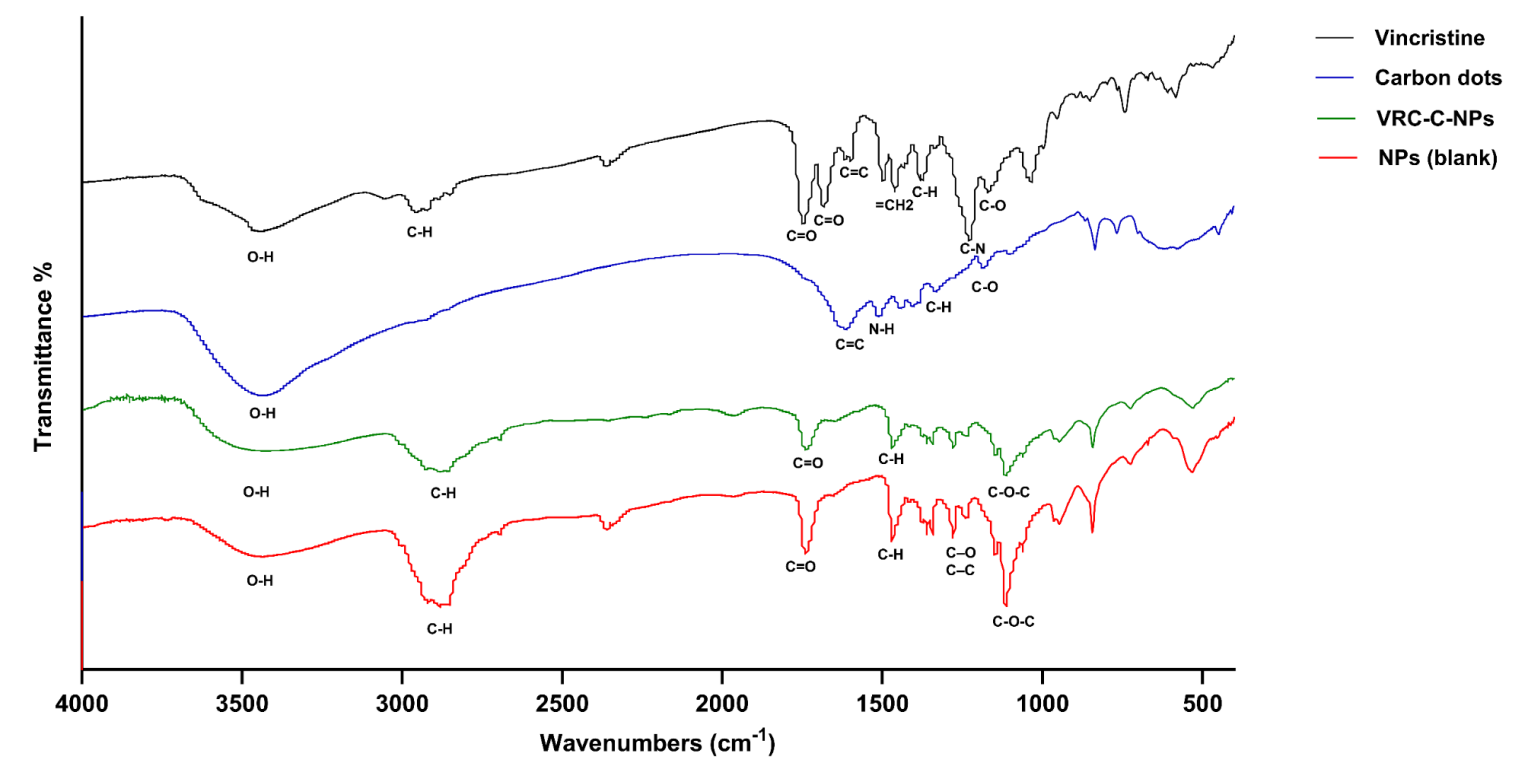
FTIR spectra of vincristine, carbon dots, VRC-C-NPs, and blank NPs.

The FTIR spectra (Fig. 4) illustrate the functional groups of vincristine sulfate, carbon dots, blank nanoparticles, and nanoparticles loaded with VRC and carbon dots. In the vincristine spectrum, a broad absorption peak is observed at 3410 cm^−1^, corresponding to O–H stretching vibrations. The peaks at 2950 cm^−1^ are due to C–H stretching vibrations. Additionally, peaks at 1747 cm^−1^ and 1690 cm^−1^ are attributed to C = O stretching vibrations, while the peak at 1230 cm^−1^ corresponds to C–N stretching vibrations and the peak at 1040 cm^−1^ represents S = O stretching vibrations. The carbon dots spectrum shows a broad absorption peak at 3440 cm^−1^ is attributed to OH groups from water molecules absorbed within the carbon dots. The peak at 1610 cm^−1^ indicates C = C bond stretching. The 1510 cm^−1^ peak signifies the presence of N-H bonds from amine groups, while the 1330 cm^−1^ peak corresponds to C-H bonds. The 1440 cm^−1^ peak suggests the presence of carboxyl groups by C-O bond vibrations. The peaks at 1180 cm^−1^ and 1000 cm^−1^ are attributed to C-O stretching and epoxy group (C-O-C) vibrations, respectively. The spectra of the blank nanoparticles and VRC-C-NPs are identical, confirming the successful encapsulation process. The nanoparticle spectrum exhibits a peak around 2865 cm^−1^ due to C-H stretching vibrations, a peak at 1747 cm^−1^ attributed to C = O stretching, a peak at 1474 cm^−1^ corresponding to C-H bending, a peak at 1485 cm^−1^ which may indicate C–O or C–C stretching, and a peak at 1113 cm^−1^ associated with C–O–C stretching vibrations.

For UV-Vis analysis, the spectra of the blank nanoparticles and VRC-C-NPs are identical, confirming the successful encapsulation process as shown in Fig. 2S (a, b), in the supplementary information, displays a single sharp peak observed at 190 nm indicating that the PCL nanoparticles are well-dispersed in solution, without evidence of aggregation, and reflect the π-π* transitions of the ester (C = O) groups^45^, which are characteristic of PCL. Additionally, the UV-Vis spectrum of PCL displays a weak shoulder around 250 nm, attributed to the n-π* transitions of the ester carbonyl group^46^. The carbon dots spectrum, depicted in Fig. 2S **(c)** in the supplementary information, shows a prominent peak emerged at a wavelength of 280 nm, attributed to the π-π* orbital transition of the carbon-carbon double bonds, and a shoulder was observed around 360 nm, corresponding to the n-π* orbital transition of bonds between carbon atoms and other functional groups containing nitrogen and oxygen atoms^47,48^. The spectrum of vincristine sulfate, shown in Fig. 2S **(d)**, in the supplementary information, exhibits maximum absorption peaks at 296 nm, 255 nm, and 219 nm^49^.

### Cell viability assay

**Fig. 5:**
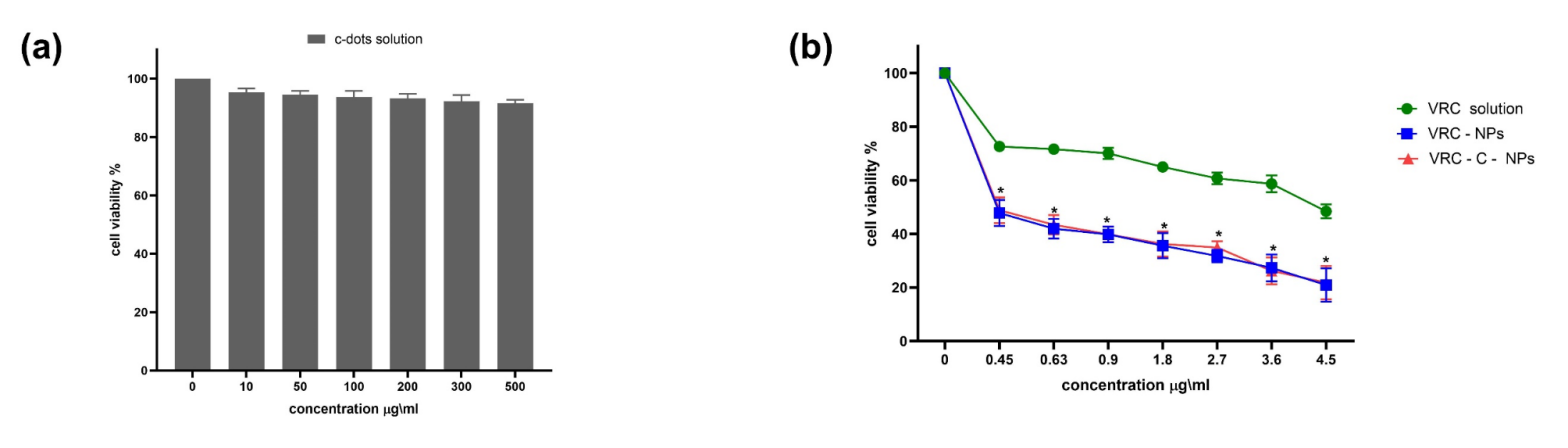
Effect of free VRC, VRC NPs, and VRC-C-NPs (**b**), and c-dots solution (**a**) on Hep-G2 cell line for 72 h. Cell viability was analyzed using the XTT assay. Data represented as mean ± SD. ^*^P value < 0.05.

The XTT assay results Fig. 5(a), showed that the c-dots solution had low toxicity even at high concentrations, up to 500 µg/ml, indicating its biocompatibility and suitability for their application in cell labeling which is effectively in line with the previous studies^15,18,21,50^. Free VRC solution inhibited cell growth with viability rates ranging from 48.4 to 72.6%, with an IC_50_ value of 4.16 µg/ml Fig. 5(b). When VRC-loaded NPs were applied, the viability decreased and ranged from 20.9 to 47.8%, with an IC_50_ value of 1.19 µg/ml Fig. 5(b). However, NPs containing both VRC and c-dots did not show a significant difference in cell viability compared to NPs containing only VRC, with viability ranging from 21.8 to 48.8%, and an IC_50_ value of 1.26 µg/ml Fig. 5(b). These results suggest that the nanoencapsulation of VRC overcomes the challenge of P-glycoprotein (P-gp) overexpression, which is a major limitation of its use in hepatocellular therapy^12,51^. This can be supported by the results of the cytotoxicity assay, as encapsulating the drug into nanoparticles enhanced its effectiveness.

### Uptake study using fluorescence microscope

**Fig. 6:**
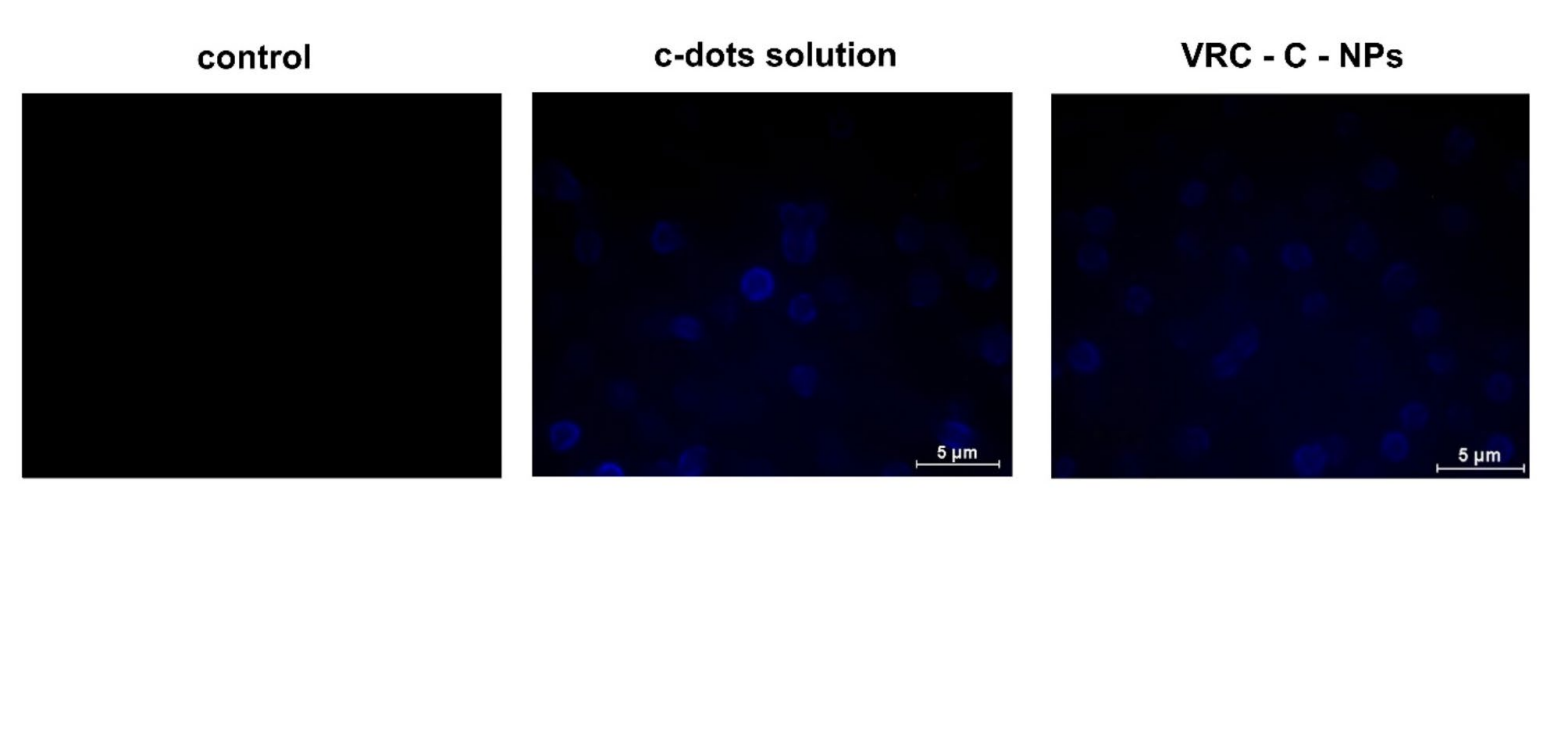
Inverted fluorescence microscopy images of cells incubated with the c-dots solution or VRC- C- NPs for 6 h.

To investigate the potential uptake of nanoparticles by cancer cells, The Hep-G2 cancer cell line was incubated with either an aqueous solution of c-dots solution or c-dots-loaded NPs, untreated cells were used as controls. Inverted fluorescence microscopy (UV filter: λ_ex_ = 390 nm; λ_em_ = 450 nm) was used to visualize the cellular uptake of NPs. The c-dots solution as well as the VRC -C-NPs were endocytosed by the cells, which was revealed by clear blue fluorescence upon excitation at a wavelength of 390 nm as shown in Fig. 6.

### Isolation of primary mouse hepatocytes and uptake studies

**Fig. 7:**
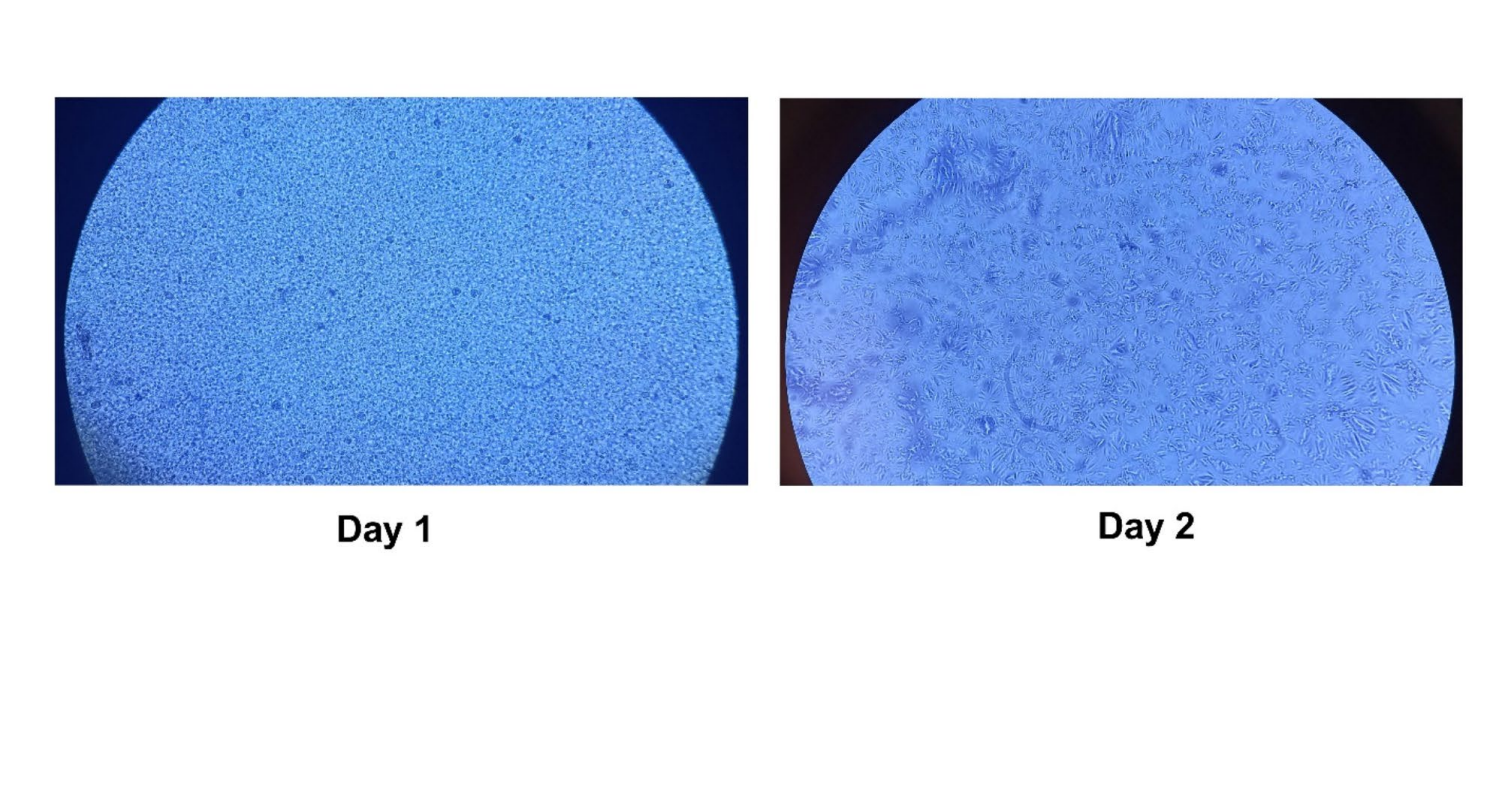
Morphology of isolated primary mouse hepatocytes.

First, the primary hepatocytes were isolated from the liver of adult male BALB/c mice. The attachment and purity of the isolated primary hepatocytes were tested. Initially, the freshly isolated hepatocytes were spherical. After one day of incubation, most hepatocytes adhered to the plate and transformed from a spherical shape to a stellate shape with multiple extensions, appearing scattered across the culture plate as shown in Fig. 7. These findings align with previous studies^52,53^.

**Fig. 8:**
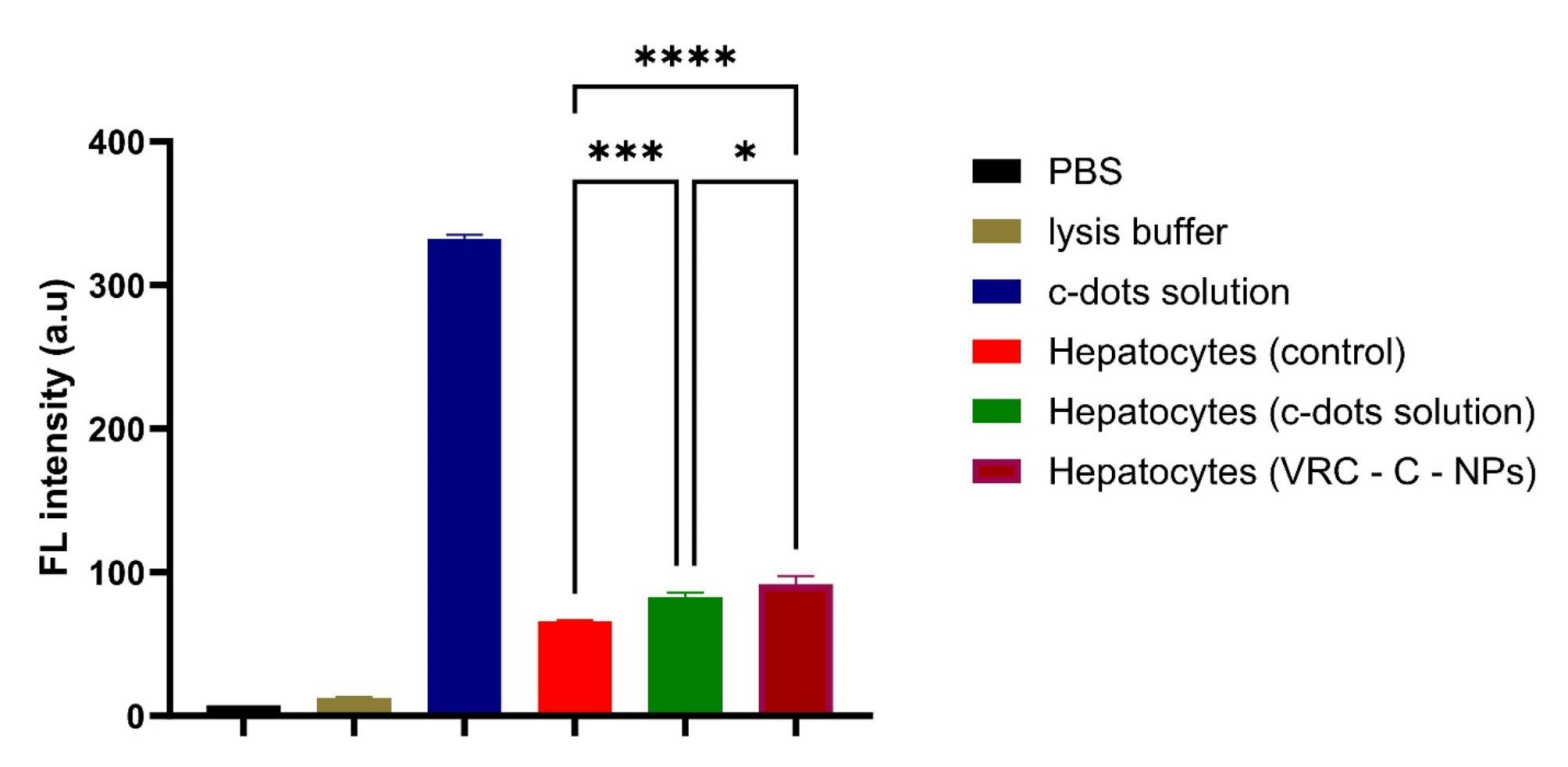
Fluorescence emission intensity recorded for isolated primary mouse hepatocytes. ^*^P < 0.05, ^***^P > 0.001 and ^****^P < 0.0001.

Next, the suspension of primary mouse hepatocytes was combined with a cell lysis buffer to dissolve the cell membrane and release the c-dots. The fluorescence intensity of the cell suspensions was then measured using a microplate fluorescence spectrophotometer (λ_ex_ = 390 nm; λ_em_ = 485 nm). The bar graph depicts the fluorescence intensity recorded from the samples (Fig. 8). Control cells exhibited autofluorescence, likely corresponding to the emission regions of Nicotinamide Adenine Dinucleotide Phosphate (NAD(P)H)^54^. However, the treated groups showed significantly higher fluorescence intensity, indicating c-dots endocytosis by the hepatocytes, with a statistically significant difference. Hepatocytes isolated from mice treated with VRC-C- NPs exhibited even higher fluorescence intensity, suggesting the targeting properties of these nanoparticles^55,56^. It is worth noting that primary hepatocytes retain many of the metabolic and structural characteristics of liver cells, leading to higher autofluorescence upon exposure to UV excitation wavelengths. In contrast, hepatocellular carcinoma cell lines have altered metabolic activities and cellular compositions^57^, resulting in lower or negligible autofluorescence under the same conditions.

## Conclusion

To effectively enhance hepatocellular carcinoma diagnosis and therapy, the current study describes a targeted vincristine delivery system and imaging probe based on carbon dots. Vincristine sulfate and c-dots were encapsulated within biodegradable polymeric nanoparticles using a double emulsion technique. Scanning electron microscopy revealed spherical PCL nanoparticles with a typical particle size of approximately 200 nm were successfully prepared and characterized. Dynamic Light Scattering (DLS) data confirmed these findings, showing a polydispersity index (PDI) of less than 0.5, indicating a uniform and homogeneous particle size distribution, and negative zeta potential values, suggesting excellent colloidal stability. The release profile of VRC-C-NPs showed a prolonged release of VRC and c-dots from the nanoparticles compared to the free solution. Moreover, the in-vitro results demonstrated that VRC-C-NPs had a more effective inhibition capacity on cancer cells than the free VRC solution. Moreover, the uptake of c-dots and VRC-C-NPs was illustrated using cancer cells demonstrating that VRC and c-dots loaded NPs exhibited higher fluorescence intensity compared to the control and free c-dots solution. Furthermore, studies on primary mouse hepatocytes showed higher fluorescence intensity in the treatment groups. Altogether, our results demonstrate the feasibility of VRC-C-NPs as novel target-specific delivery carriers for the treatment of liver cancer and open new avenues for cancer cell imaging.

## Materials and methods

### Chemicals and reagents

Vincristine sulfate (VRC) was purchased from Guangzhou Hanfang Pharmaceutical Co. Ltd, China, Polycaprolactone (50 kDa), Poloxamer 407, Span 60, dialysis bag molecular weight cut-off (12 kDa), Fatal Serum Albumin, Penicillin-Streptomycin Solution Hybri-Max™, Dulbecco’s Modified Eagle’s Medium (DMEM), Poly-L-lysine, Collagenase V, Hanks’ Balanced Salt Solution (HBSS), Paraformaldehyde and HEPES, were all obtained from Sigma Aldrich, UK. Roswell Park Memorial Institute (RPMI) from Euro Clone, Italy. Trypsin powder from PAN-Biotech, Germany, and Heparin solution 5000 UI/ml from PANPHRMA, France. Other chemicals used were of analytical purity.

### Method of VRC and c-dots quantification

VRC and c-dots were assayed by HP-TLC. Vincristine was calibrated using the method parameters detailed in our previous study^42^. For carbon dots assay the samples were spotted mechanically in a volume of 20 µl, 1.2 cm apart and 1 cm from the bottom of the silica gel-coated aluminum TLC plate (CAMAG automatic TLC sampler 4, Germany), The plate was then developed in [ethyl acetate (60), methanol (30), deionized water (15), and formic acid (1)] (V/V), and the fluorescence intensity was measured at 366 nm (Hg lamp, measurement mode: Fluorescence) using the HP-TLC scanner (CAMAG TLC scanner 3, Germany).

### Preparation of the VRC and c-dots loaded PCL nanoparticles

Carbon dots were synthesized following the method described in a previous study^48^. Nanoparticles were prepared using a water-in-oil-in-water emulsion method^42,58^. Vincristine sulfate 500 µg and carbon dots 1 mg were dissolved in 500 µl of phosphate-buffered saline (pH 7.4) and then added to an organic solution containing (10-20-30) mg of PCL and 0.12 g of Span 60 in 6 mL of dichloromethane. This mixture was emulsified using an Ultra-Turrax T 18 basic (IKA^®^ Labotechnik, Staufen, Germany) at 25,000 rpm for 5 min to form a primary water-in-oil emulsion. The primary emulsion was then added to another aqueous solution containing 50 ml of phosphate-buffered saline (pH 7.4) and 0.12 g of poloxamer 407 and emulsified under the same conditions to prepare the double emulsion Table 2. The organic solvent was removed using a rotary evaporator (Buchi, Switzerland) at 72 mbar and 40 °C. The aqueous phase was concentrated to a final volume of 10 ml. The emulsion was then placed in dialysis bags (MW 12 kDa) and dialyzed against 200 mL of water for 2 h at room temperature to remove unencapsulated vincristine sulfate and carbon dots.

**Table 2:** Formulations of vincristine sulfate and carbon dots loaded polycaprolactone nanoparticles prepared by the double emulsion method.

Formulation	VRC (mg)	c-dots (mg)	PCL (mg)	DCM (ml)	Span 60 (inner phase)	Poloxamer 407
VRC -C- NPs 1	0.5	1	10	6	2%	0.2%
VRC -C- NPs 2	0.5	1	20	6	2%	0.2%
VRC -C- NPs 3	0.5	1	30	6	2%	0.2%

### Characterization of the PCL nanoparticles

Dynamic light scattering (DLS) analysis was employed to measure the average dynamic size, PDI, and zeta potential measurements. Scanning Electron Microscopy (SEM, TESCAN, VEGA, Czech Republic) was used to examine the size, shape, and surface morphology of nanoparticles. Encapsulation efficiency was measured using the direct method. The samples were analyzed using HP-TLC, considering the analytical methods used for both vincristine and carbon dots. The EE% was calculated using the following equation:

$${\text{\% }}\;{\text{Encapsulation}}\;{\text{efficiency = (Actual}}\;{\text{loaded}}\;{\text{drug /Theoretical}}\;{\text{loaded}}\;{\text{drug) }}$$

The dialysis method was employed to study the release profile^59,60^. Nanoparticles were placed in dialysis bags with a molecular weight cut-off of 12,000 Daltons and dialyzed against 50 ml of deionized water. The release experiment was performed in an incubator shaker set at 125 rpm and 37 °C. The release medium was collected and refreshed at each time point. Subsequently, all samples were dried completely under low pressure using a rotary evaporator until completely dry. The dried samples were analyzed using HP-TLC. The data were presented as an accumulation curve, illustrating the release profile over time.

### Fourier transform infrared spectroscopy

The Fourier transform infrared (FTIR) spectra of vincristine sulfate, carbon dots, blank nanoparticles, and VRC and carbon dots loaded nanoparticles were analyzed using Nicolet™ FT-IR spectrometer (Thermo Scientific™, USA). The samples were mixed with potassium bromide (KBr) at a 2% w/w ratio. The resulting mixture was finely ground into a powder and then compressed into KBr discs using a hydraulic press at 10,000 psi. Each disc was analyzed by scanning across a wavenumber range from 4000 cm^−1^ to 400 cm^−1^. The characteristic peaks corresponding to different functional groups in the samples were recorded.

### Spectroscopic scanning using UV-Vis spectrophotometer

The absorption spectra of vincristine sulfate, carbon dots, blank nanoparticles, and nanoparticles loaded with VRC and carbon dots were analyzed using UV-Vis spectroscopy (6850 UV/Vis Spectrophotometer - JENWAY) across the wavelength range of 190 to 400 nm. This analysis aimed to evaluate the optical characteristics of both the carbon dots and vincristine sulfate, given their UV absorption properties. Additionally, UV-Vis spectroscopy was used to assess the optical properties of the nanoparticles, providing complementary information to other methods used for determining their size and shape^61^.

### In vitro toxicity study

The principle of the method is measuring cell metabolic activity by colorimetric titration using XTT dye. The NADH-dependent cellular oxidoreductase enzyme, which indicates the number of living cells, converts the water-soluble yellow tetrazolium dye into the orange water-soluble formazan pigment through mitochondrial dehydrogenase activity^62^.

### Cell viability assay

The Hep-G2 cancer cell line was purchased from the Cell Bank of the Atomic Energy Commission, Department of Molecular Biology and Biotechnology. The cells were cultured in a 96-well microplate in RPMI-1640 medium supplemented with penicillin (100 IU/ml), streptomycin (100 mg/ml), and 10% fetal calf serum (FBS), at a cell density of 20,000 cells/well. The cells were incubated for 24 h at 37 °C with 5% CO_2_. After incubation, the culture medium was removed and replaced with a new complete medium containing various concentrations of carbon dots (10–50 – 100–200 -300–500 µg/ml) and vincristine sulfate solution, VRC loaded NPs, and VRC and c-dots loaded NPs (0.45, 0.63, 0.9, 1.8, 2.7, 3.6, 4.5 µg/ml). Untreated wells were used as a negative control. The plates were incubated for 72 h at 37 °C with 5% CO_2_. After the treatment period, the XTT reagent was added to the culture medium. The plate was then incubated at 37 °C with 5% CO_2_ for 4 h. Optical absorption was measured at a wavelength of 450 nm using a microplate spectrophotometer (BioTek, Epoch, UK). The percentage of cell viability was calculated using the following equation:

$${\text{Viability}}\:\% {\text{ = }}({\text{Absorbance}}\:{\text{of}}\:{\text{Sample/Absorbance}}\:{\text{of}}\:{\text{Control}})\: \times \:{\text{100}}$$

The results were also expressed as the 50% cell growth inhibitory concentration (IC_50_).

### Uptake study using fluorescence microscope

The Hep-G2 cancer cell line was plated in a 6-well tissue culture plate at a density of 2 × 10^6^ cells/well and incubated at 37 °C with 5% CO_2_ for 24 h. After incubation, the culture medium was removed and replaced with a new complete medium containing the aqueous solution of carbon dots (200 µg/ml) or c-dots loaded nanoparticles and incubated for 6 hours. After the incubation period, the cells were washed three times with phosphate buffer to ensure the complete removal of any unbound carbon dots. The cells were then fixed with 2.5% formaldehyde solution for 10 min and subsequently washed with PBS. The uptake of nanoparticles by the cells was assessed using an inverted fluorescence microscope, based on the characteristic fluorescence of the carbon dots. Observations were conducted using an inverted fluorescence microscope (Nikon ECLIPSE TS 100, Japan).

### Isolation of primary mouse hepatocytes

Male BALB/c mice were obtained from the Experimental Animal Unit of the Atomic Energy Commission. Each mouse weighs approximately 20 g. All animal procedures were performed in accordance with the Guidelines for Care and Use of Laboratory Animals of Breeding Unit for Mice at the Department of Molecular Biology and Biotechnology, Atomic Energy Commission of Syria, and approved by the Animal Ethics Committee of Damascus University which aligned with the ARRIVE guidelines^63^ and relevant regulations.

Euthanasia was performed using an approved method as defined by the American Veterinary Medical Association (AVMA) Guidelines for the Euthanasia of Animals (2020)^64^. The animals were euthanized with an anesthetic solution of ketamine and xylazine (87.5 and 12.5 mg/kg body weight, respectively) administered via intraperitoneal injection.

The mice were housed in groups under standard laboratory conditions, including a 12-hour light/dark cycle, a temperature of 22 ± 2 °C, and a relative humidity of 55 ± 5%. They had access to adequate food and water throughout the study.

The study groups were divided into three groups, each containing three mice. The first group received an intraperitoneal injection of an aqueous solution of carbon dots at a concentration of 100 µg/ml. The second group was administered VRC and carbon dot-loaded nanoparticles containing 180 µg /ml vincristine and 100 µg/ml carbon dots. The control group was left untreated.

One hour after administration, the mice were anesthetized with an intraperitoneal injection of an anesthesia mix composed of ketamine (87.5 mg/kg) and xylazine (12.5 mg/kg). Liver perfusion was performed by pumping the Perfusion Buffer (Ca^2+^ and Mg^2+^ free HBSS, 10 mM HEPES buffer,100 U/ml penicillin G, and 100 mg/ml streptomycin, heparin 8 UI/ml, ) at a constant rate of 4.5 ml/min through the portal vein using a 24 G intravenous catheter. Immediately upon the appearance of white spots, the inferior vena cava (IVC) was cut with scissors to let the blood out. The perfusion process continued until the liver turned pale. The liver was excised and placed in a nutrient medium (DMEM, 10% v/v fetal bovine serum, 100 U/ml penicillin G, and 100 mg/ml streptomycin) in an ice bath to maintain cell viability. The liver was mechanically dissected by tearing it into pieces with tips and forceps. The tissue fragments were placed in a tube containing an enzyme mixture of trypsin (250 U/mg) and collagenase V (359 U/mg) dissolved in the nutrient medium at a ratio of 10 ml of enzyme mixture per gram of tissue and incubated at 37ºC with 5% CO_2_ for 30 min. The cell suspension was passed through a 70 μm cell strainer into a clean, sterile 50 ml tube containing 20 ml of DMEM medium. The cell suspension was centrifuged at 300 x g for 10 min. The supernatant was discarded, and the cells were resuspended in a DMEM medium. This process was repeated three times. Cell viability was assessed using a 1:1 dilution of trypan blue, and to examine the morphology of the isolated primary mouse hepatocytes, the cells were then seeded (25,000 cells/cm²) in poly-L-lysine (10 mg/ml) coated T25 flasks. The cultures were incubated at 37 °C in a humidified chamber with 5% CO_2_, and the culture medium was changed 24 h after seeding^39,52^.

### Uptake study using fluorescence spectroscopy

To study the uptake of nanoparticles or carbon dots by hepatocytes, fluorescence intensity was measured for the cell suspension from mice administered either the aqueous solution of carbon dots or vincristine and carbon dots loaded nanoparticles. A 500 µl aliquot of the cell suspension was mixed with a lysis buffer (pH = 10) containing (2.5 M) NaCl, (0.1 M) EDTA, (1.2) M Tris-base, (0.2 M) NaOH, and (0.03408 M) SDS. The mixture was incubated for 10 min to ensure cell membrane disruption^15^. Phosphate buffer was used as a negative control, and the carbon dots solution was used as a positive control. Fluorescence intensity was measured using a fluorescence microplate reader (Thermo Scientific™, USA).

### Statistical analysis

Statistical difference analyses were assessed through one-way ANOVA with the Tukey-Kramer post-test using GraphPad Prism (10.1.2). The data are presented as mean ± SD. P values less than 0.05 were considered significant.

## Electronic supplementary material

Below is the link to the electronic supplementary material.

Supplementary Material 1

## Data Availability

The data used to support the findings of this study are available from the corresponding author upon request.
