# Supplementary material for: Polymeric nanoparticles loaded with vincristine and carbon dots for hepatocellular carcinoma therapy and imaging
Source: Sci Rep. 2024 Oct 18;14:24520. doi: 10.1038/s41598-024-75332-1 (PMC11489775; doi:10.1038/s41598-024-75332-1)
Supplement: Supplementary file 1 — Supplementary Material 1 [file 41598_2024_75332_MOESM1_ESM.docx]

**Polymeric Nanoparticles Loaded with Vincristine and Carbon Dots for Hepatocellular Carcinoma Therapy and Imaging**

Walaa Fawaz^1*^, Abdulsamie Hanano ^2^, Hossam Murad^2^, Amal Yousfan^1,3^, Ibrahim Alghoraibi^4^, and Jameela Hasian^1,5^

^1^Department of Pharmaceutics and Pharmaceutical Technology, Faculty of Pharmacy, Damascus University, Damascus, Syria.

^2^Department of Molecular Biology and Biotechnology, Atomic Energy Commission of Syria (AECS).

^3^Department of Pharmaceutics and Pharmaceutical Technology, Faculty of Pharmacy, Al Andalus University for Medical Sciences, Tartus, Syria.

^4^Department of Physics, Faculty of Science, Damascus University, Damascus, Syria.

^5^Department of Pharmaceutics and Pharmaceutical Technology, Faculty of Pharmacy, Yarmouk Private University, Damascus, Syria.

*Address correspondence to this author at the Department of Pharmaceutics and Pharmaceutical Technology, Faculty of Pharmacy, Damascus University, Damascus, Syria; Tel/Fax: 00 963 11 2131871, 00 963 11 2119837;
E-mails: fwalaa33@gmail.com, walaa.fawaz@damascusuniversity.edu.sy.


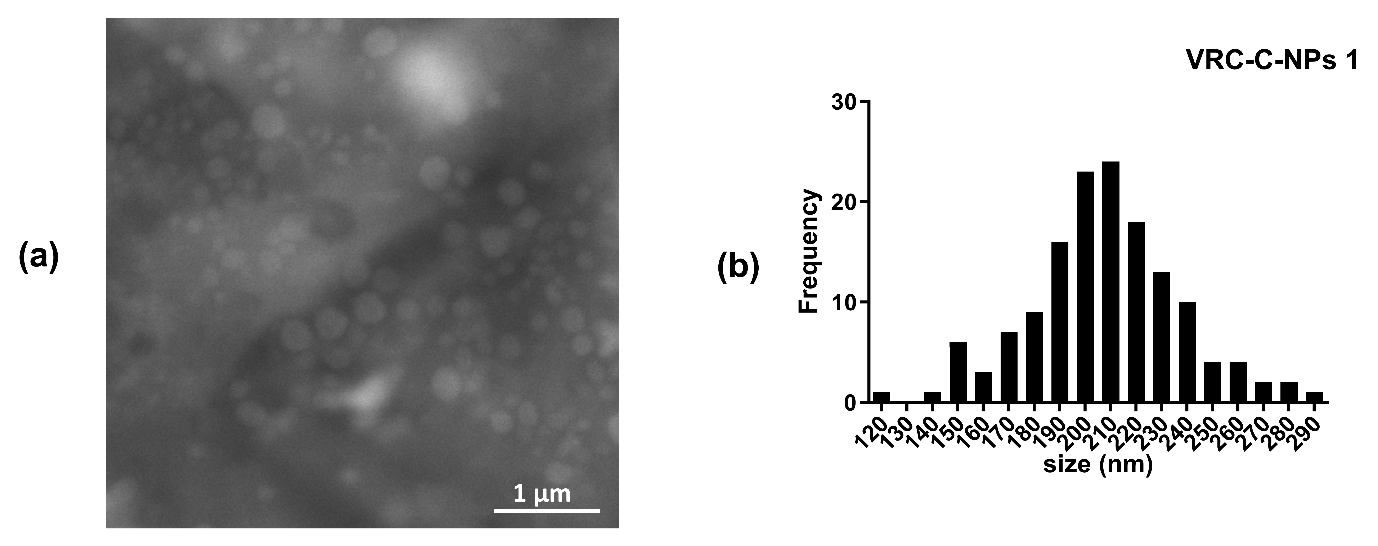


**Figure (1S).** SEM micrograph of nanoparticles (a), and SEM size distribution histogram (b), after incubation with Human Serum Albumin HSA (20%) for 6 hours at 37°C.

**
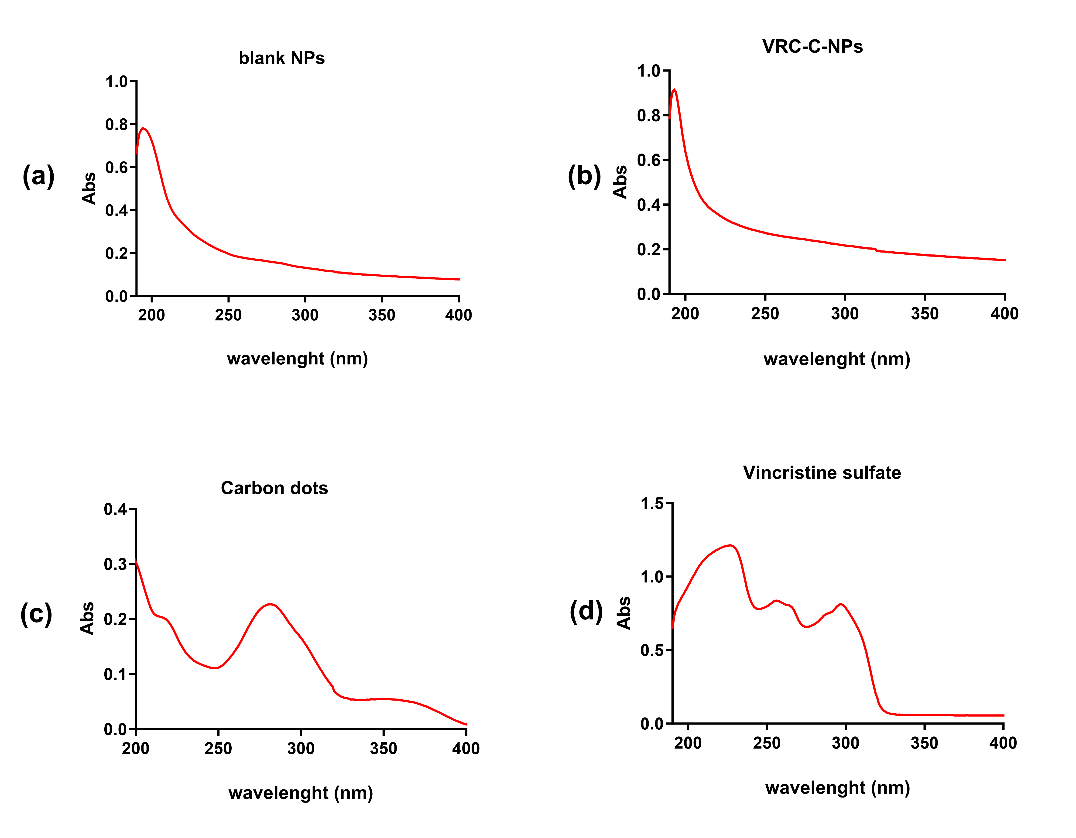
**

**Figure (2S).** UV-Vis absorption spectra of blank NPs (a), VRC-C-NPs (b), Carbon dots (c), and Vincristine sulfate (d).
